# The effectiveness of the Allen Carr smoking cessation training in companies tested in a quasi-experimental design

**DOI:** 10.1186/1471-2458-14-952

**Published:** 2014-09-13

**Authors:** Arie Dijkstra, Rixt Zuidema, Diederick Vos, Marike van Kalken

**Affiliations:** University of Groningen, Grote Kruisstraat 2/1, 9712 TS Groningen, The Netherlands; University of Nijmegen, Nijmegen, The Netherlands; Jobsites Inc., Amsterdam, Amsterdam, UK; Bewegen Werkt Holding B.V., Enschede, The Netherlands

**Keywords:** Smoking cessation, Allen Carr training, Quasi-experimental, CO validation

## Abstract

**Background:**

The Allen Carr training (ACt) is a popular one-session smoking cessation group training that is provided by licensed organizations that have the permission to use the Allen Carr method. However, few data are available on the effectiveness of the training.

**Methods:**

In a quasi-experimental design the effects of the existing practice of providing the ACt to smokers (n = 124) in companies on abstinence, were compared to changes in abstinence in a cohort of similar smokers in the general population (n = 161). To increase comparability of the smokers in both conditions, smokers in the control condition were matched on the group level on baseline characteristics (fourteen variables) to the smokers in the ACt. The main outcome measure was self-reported continuous abstinence after 13 months, which was validated using a CO measurement in the Act condition.

**Results:**

Logistic regression analyses showed that when baseline characteristics were comparable, significantly more responding smokers were continuously abstinent in the ACt condition compared to the control condition, Exp(B) = 6.52 (41.1% and 9.6%, respectively). The all-cases analysis was also significant, Exp(B) = 5.09 (31.5% and 8.3%, respectively).

**Conclusion:**

Smokers following the ACt in their company were about 6 times more likely to be abstinent, assessed after 13 months, compared to similar smokers in the general population. Although smokers in both conditions did not differ significantly on 14 variables that might be related to cessation success, the quasi-experimental design allows no definite conclusion about the effectiveness of the ACt. Still, these data support the provision of the ACt in companies.

## Background

Allen Carr’s “Easyway to Stop Smoking training” (ACt) is an one-session smoking cessation group training that is provided by licensed organizations that have the permission to use the Allen Carr method. The method also has been published widely in book form. The Allen Carr website states they have: “… sold over 10 million stop smoking books in 57 countries in more than 38 languages […] Every year Allen Carr’s Easyway To Stop Smoking Clinics and online quit smoking programmes cure over 50,000 smokers. There are stop smoking seminars in more than 150 cities in over 45 countries worldwide” [1] In the Netherlands, the licensee holder of the ACt primarily provides the training through companies, exposing about 1,500 smokers to it each year.

However, few data are available on the effectiveness of the training. Only percentages of abstinence from cohorts of smokers who followed the training are published. One study [2] reported that of 357 smokers exposed to the ACt in a company, at least 40% reported point-prevalence abstinence after 12 months, while in the cohort study [3] (N = 510) 51% of the smokers reported abstinence about 3 three years after the ACt. Two other studies in AC smoking cessation clinics reported percentages abstinence up to 26% [4]. Thus, although the ACt seems widely used, very limited evidence is available with regard to its effectiveness.

The best scientific design to test whether the ACt method incorporates effective ingredients is the RCT. For example, recruited smokers might be randomly assigned to an ACt condition or to a no-intervention control condition. However, this is not always possible. In the present study we aimed to assess whether providing the ACt to smokers in companies is effective in stimulating abstinence from smoking. This implies that the test of effectiveness must be conducted within the setting of the companies, taking into account the demands and desires of companies that join the study. For example, once companies are interested in the ACt, they want to be certain that they can offer the ACt to their smokers, and they find it less desirable to let their smokers be randomized or to be randomized as a (department of) a company to the ACt condition or a control condition. Therefore, we tested the ACt in companies in a quasi-experiment [5]. In a quasi-experiment the practice of the ACt is monitored and the changes are compared to the changes in an independently recruited cohort of smokers, implying that no randomization took place. Therefore, the quality of such a quasi-experimental design primarily depends on the baseline similarity on relevant variables of the participants in the ACt group and those in the control group. Therefore, in the present study, the most important demographic variables (e.g., age, gender), smoking behavior related variables (e.g., FTND), and psychological variables (motivation to quit, self-efficacy) were assessed at pretest to test and shape the similarity.

The composition of the control group further determines the exact research question the quasi-experiment will address. In the present study of ACt in companies, an independent control group might be recruited from other (similar) companies but also from the general population. When the control group is recruited from other companies, the design will show whether it is fruitful to expose smokers in companies “to the ACt”. When the control group is recruited from the general population, the design will show whether it is fruitful to expose smokers “in companies to the ACt”. Thus, in this case the treatment package is “ACt in companies”. This is what the present study is about: A quasi-experiment in which smoking employees in companies are exposed to the ACt, while their abstinence rate assessed after 13 months is compared to that of baseline-matched smokers recruited from the general population.

## Methods

### Recruitment

The company “Bewegen Werkt” [Exercise Works] markets the ACt in companies. Participants in the experimental group were recruited between January 2011 and May 2012 through companies that decided to purchase the ACt for their smoking employees. Smokers could follow the training for free. Smokers were first informed by their Human Resource department about an informative session preceding the actual ACt that was going to take place. This informative session was about the ACt but it was also mentioned that some scientific measurements would take place. At the end of this session interested smokers could leave their contact information. They were then assigned to an ACt trainer who was given access to the e-mail addresses of the smokers. These addresses were used by the researchers to approach the smokers about two weeks before the ACt was given. This e-mail emphasized that their participation in the study was voluntary and that they could withdraw from the study at any time without penalty. In the e-mail the (three) measurements were announced. About one week after they received this information e-mail, another e- mail with the link to the baseline (pretest) on-line questionnaire (T1) was sent.

Participants in the control group were recruited between January 2011 and April 2012 through general mass-media and social media throughout the Netherlands. Advertisements, presented to over 100 regional newspapers (no records were kept on actual ad placements) and to social media, invited smokers “who wanted to quit” to join a study on smoking and smoking cessation that would consist of filling out three times an on-line questionnaire. Interested smokers could e-mail to the address mentioned in the advertisement and they were subsequently sent an information e-mail with the same information as the smokers in the experimental group received. One week after that, an e-mail with the link to the baseline on-line questionnaire (T1) was sent.

### Procedure

Via an informed consent option in the online questionnaire, participants gave their permission for participation. Participants in both conditions were not informed about the existence of the other condition, only about their own condition. Between January 2011 and May 2013 we sent three times an e-mail with a link to an online questionnaire. The baseline questionnaire (T1) was sent about one week before the ACt started. The follow-up questionnaires were sent 2 weeks after the training took place (T2) and 13 months after T2 (T3). In the control group, smokers received the T2 and T3 on-line questionnaires, 2 weeks and 14 months after T1, respectively. Except for the T1 measurement in the experimental group, three reminder e-mails were sent concerning each measurement: One after three days, one after a week and the final one after two weeks. To schedule the CO-measurement, the participants in the experimental condition who reported on T3 to be abstinent were contacted. It was tried to schedule the CO-measurement within three weeks after the participants filled in the T3 questionnaire.

Financial compensation was offered to lower dropout rates. Participants were informed in the first e-mail that they would earn 12.50 euros when they filled in the T1 measurement and another 20 euros when they would complete the study by also filling out the T3 measurement. In the mail on scheduling the CO-measurement, participants were also informed that they would receive 20 euros, whatever the result of the test was. The research was approved by the Ethical Committee Psychology of the University of Groningen.

### Allen Carr’s Easyway to Stop Smoking training

The ACt consisted of a one-meeting training including 5 to 8 smokers led by a trainer who was an ex-smoker. Trainers are trained to provide the ACt, following a loosely protocolled scheme in which they provide the trainees subsequently with different questions and answers (the Allen Carr manual containing the protocol has not been made publicly available by the license holder). When analyzing the content of the training, the following working mechanisms can be recognized: The core of the argument to quit smoking is that smoking tobacco has no real benefits; it is only smoking away withdrawal symptoms in an addicted body. From this premise it follows that when the body is no longer addicted, smoking has no beneficial effects whatsoever. This core idea is repeated in different words using different analogues. Beliefs and experiences of the trainees regarding the benefits of smoking are restructured and challenged against the background of this notion. The aim is to make the smokers completely endorse the core idea, thereby fundamentally changing their perspective on their smoking behavior, including reappraising their past experiences with smoking. This restructuring of the beliefs on the benefits of smoking can be conceptualized as an expectancy challenge, as is applied to lower alcohol consumption in several studies [6–8]. In smoking cessation, very few data are available on the effects of expectancy challenge [9, 10]. Besides the expectancy challenge, other potential working mechanisms can be recognized in the ACt. One effect of lowering the perceived benefits of smoking is that the task of smoking cessation becomes easier. This may lead to a relative increase in self-efficacy expectations, which is a reliable predictor of abstinence [11, 12]. Furthermore, the trainer always is an ex-smoker, which makes him a model. In addition, the group process may support a climate of change. Importantly, in the ACt protocol there is no explicit room for motivating the trainees to quit. From the protocol and from observation it seems that the only motivating information in the training comes from the positive feelings that are associated with no longer feeling addicted and having to smoke, and taking control over one’s life. Thus, the ACt does not try to motivate smokers to quit, it rather tries to lower the motivation to smoke.

After the training, participants were in the opportunity to call their trainer by telephone for support to stay abstinent. Furthermore, they could visit a follow-up training (on their own initiative) for free in the year after their training. As this was part of the package of the ACt, no data were gathered on the use of these facilities.

### Biochemical validation

To validate the self-reported abstinence, a CO-measurement was conducted among respondents who indicated to be abstinent at T3. These T3 respondents were contacted by mail to make an appointment. The researchers would visit the company site and conduct the CO- measurement. This measurement was protocolled to take place in a sitting position, in a private room after the T3 abstinence was verified again by self-report. When the CO-measurement indicated a higher than expected CO-level, some additional questions were asked to check the reasons for the high level. The CO-level was considered to verify the report of abstinence when it was below 10 ppm, and when it was 10 ppm or higher it was considered to falsify the abstinence [13]. The CO-measure apparatus was the piCO **+** Smokerlyzer**®**. Before the measurements started, the apparatus was calibrated by the company from which it was purchased.

### Measures

The baseline questionnaire consisted of different chapters. Firstly, demographics were assessed: gender, age, and level of education. Secondly, smoking behavior was assessed with a question on the number of cigarettes smoked a day, and another five questions that together comprise the FTND test. [14] Thirdly, the baseline questionnaire contained measures regarding the psychology of smoking and quitting smoking: The pros of quitting, the pros of smoking and self-efficacy expectations.

The pros of quitting assess the motivation to quit. They refer to the reasons for smokers to quit and because they are related to personally valued outcomes they provide the energizing power that underlies behavior change. The pros of quitting were assessed using four short scales of each three items on the following topics: The expectations concerning positive long-term physical consequences of quitting (e.g., “Quitting lowers my risk for lung cancer”), expectations concerning positive short-term physical consequences of quitting (e.g., “Quitting increases my physical stamina”), expectations concerning positive social consequences of quitting (e.g., “Quitting makes me a better model for others”), and expectations concerning the positive self-evaluative consequences of quitting (e.g., “Quitting makes me feel better about myself”). The scales were shown to be robust predictors of future quit attempts (during 9, 7, and 6 months intervals) in three independent samples of smokers [14]. The items were in the following format: “Quitting smoking [leads to the positive consequence] ....”, and could be scored from “not sure” or “not expecting a certain outcome” (0) to a “strong expectation of the outcome” (3). The mean item scores were used as the scale scores. The Cronbach’s α’s of the four scales were .88, .64, .71, and .69, respectively.

The pros of smoking assess the motivation to smoke or the level of “psychological addiction”. They refer to the reasons to smoke; the expectation that smoking will have specific valued effects. The pros of smoking were assessed using a 9 item scale on the cognitive functions of smoking, such as weight regulation, relaxation, and coping with anger, and a 5 item scale on the positive affective experiences of smoking, for example, satisfying, likable and pleasant. The items of the first scale were validated in earlier studies [15, 16]. The second scale was developed for this study. The items were in the following format: “Smoking helps to/Smoking is to me ....”, and could be scored on a 5-point scale with the options: “completely disagree” (1), “disagree a little” (2), “not disagree/not agree” (3), “agree a little” (4), “completely agree” (5). The mean item scores were used as the scale scores (Cognitive pros α = .80; affective pros α = .82).

Self-efficacy expectations assess the confidence smokers have that they are able to remain abstinent. This confidence determines the effort and persistence smokers invest in their smoking cessation. Self-efficacy expectations were assessed using three short scales that were validated in earlier studies [17, 18]. The items assessed the level of confidence of each participant in his or her ability to refrain from smoking in emotional (“When you feel angry”), social (“When you are offered a cigarette”), and habitual (“After diner”) situations. All items were measured on a 7-point scale and could be scored from “not at all sure I am able to” (-3) to “very sure I am able to” (+3). The mean item scores were used as the scale scores. The α’s of the three scales were .92, .87, and .86, respectively.

Because we do not report data from the first posttest questionnaire (T2) that was administered three weeks after the first one, this measurement will not be presented here.

The second posttest questionnaire (T3) was administered 14 months after the pretest and it assessed smoking behavior since the ACt (in the experimental group) or since the first posttest (in the control group). In the experimental group, self-report abstinence was assessed with two subsequent questions. The first abstinence question was: “Did you quit smoking immediately after the ACt?” The answering categories were: “Yes, and I still do not smoke”; “Yes, but later I started smoking again”; “No”; “I do not remember”. When respondents chose the first option, they were assumed to be continuous abstinent according to their self-report. When respondents in the experimental group chose another option, they were routed to the second abstinence question: “Have you been smoking since the second measurement about 13 months ago (even one cigarette)?”, “Yes”/”No”. In the control group this question was the primary abstinence question. Respondents who answered “No” were considered continuous abstinent according to their self-report. Respondents who answered this question with “Yes” were asked subsequent questions on their smoking behavior during the past 13 months.

The use of smoking cessation support since the second measurement (which was administered three weeks after the baseline measurement) was assessed with one categorical question: Respondents could score one category from the following six: 1. nicotine replacement/pharmacological support of any kind; 2. (individual) counseling/coaching (including e.g. acupuncture or support from the GP); 3. self-help materials; 4. nicotine replacement/pharmacological support and coaching; 5. none of the above; 6. all of the above.

## Results

### Response and attrition

**Figure 1:**
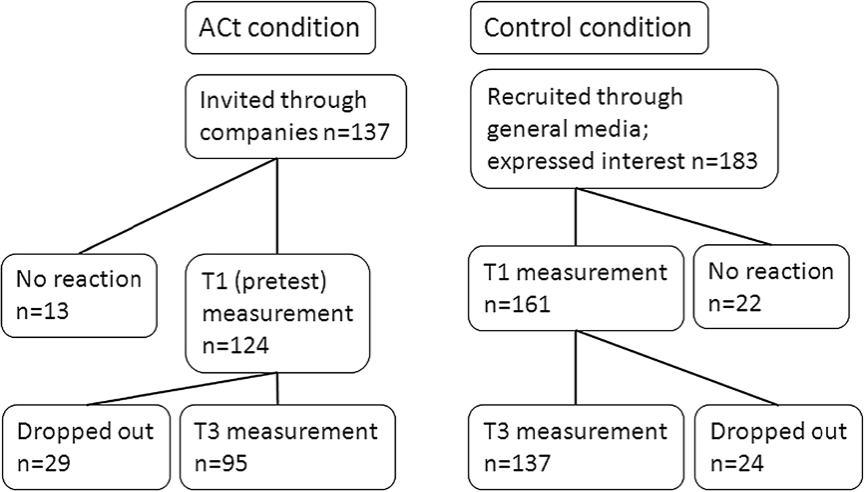
**The attrition figures in the two conditions in the quasiexperimental design.**

In the experimental group a total of 137 smokers in 15 companies in 19 training groups were trained. Of these smokers who were all invited to join the study, 124 filled in the T1 measurement. Of these T1 respondents, 95 also filled in the T3 measurement. Of the 183 smokers in the control group who expressed interest to join the study, 161 filled in the T1 measurement. Of these T1 respondents, 137 also filled in the T3 measurement (see Figure 1).

Thus, the baseline questionnaire was filled in by 285 smokers (124 in the ACt condition, 161 in the control condition). Of these smokers, 232 also provided long term data (81.4%), leaving 95 in the ACt condition (76.6%) and 137 in the control condition (85%). A Chi-square test on these drop out percentages showed a trend towards a significant difference (Pearson’s Chi-square p = .068).

In an attrition analysis, smokers who did not fill in the T3 questionnaire (drop outs) were compared to those who did fill in the T3 measurement on 14 variables: gender, level of education, age, number of cigarettes smoked a day, FTND score, the pros of quitting (four scales), the pros of smoking (two scales), and self-efficacy (three scales). Univariate analyses of variance on the continuous variables showed that those who dropped out, firstly, scored significantly higher on physical dependence (FTND), F(283,1) = 4.71, p = .031; respondents M = 3.83 (SD = 2.28) versus drop outs M = 4.59 (2.3). Secondly, drop outs scored significantly lower on self-efficacy in social situations, F(282,1) = 3.99, p = .047; respondents M = 4.31 (SD = 1.36) versus drop outs M = 3.89 (1.32). For the two categorical variables, gender and level of education, the Chi-square analyses showed that drop outs did not differ significantly from respondents.

### Smoking cessation support

Of the 208 respondents who answered the T3 question about smoking cessation support since the second measurement, 20 stated to have used nicotine replacement/pharmacological support of any kind, 11 had received counseling/coaching, 5 used self-help materials, 1 reported to have used all types of support, 4 reported to have received the combination of nicotine replacement/pharmacological support and counseling/coaching or self-help, and 167 indicated to not have used any of support. To check whether the respondents in the Act condition and the control condition differed in the support they had received, the categories were recoded into one dichotomous variable: no support (n = 167) versus any support (n = 40). A Chi-square analysis showed that that the difference was significant, *X* [2] (1, *N* = 208) = 13.15, *p* < .001: 7.9% of the respondents in the Act condition received support, against 28% in the control condition. In the Act condition, only 2 respondents used nicotine replacement/pharmacological support.

### Analyses in respondents

#### Comparability and matching in respondents

The baseline comparability of respondents in the Act condition and the control condition was tested using the above 14 variables that could be related to smoking abstinence. Using univariate analyses (ANOVA for continuous variables and Chi-square for categorical variables), there were significant differences only on the affective pros of smoking, F(224,1) = 5.44, p = .021, and on self-efficacy with regard to social situations, F(224,1) = 5.81, p = .017: Smokers in the ACt condition scored higher on the affective pros (ACt condition M = 3.87 (SD = 0.76); control condition 3.61 (SD (0.89)), and higher on the measure of self-efficacy (ACt condition M = 4.56 (SD = 1.28); control condition M = 4.13 (SD (1.4)). Thus, smokers in the control condition were less psychologically dependent but had also less confidence that they would be able to quit smoking.

To increase the comparability of the conditions, a matching procedure was applied in which the 10% respondents with the most extreme scores were removed from the control condition, leading to the following decision. To increase the mean affective dependence of the control condition, the smokers with the lowest 10% scores on the affective pros were removed. In addition, to increase the mean level of confidence, the smokers with the lowest 10% scores on the affective pros were removed. This left 114 respondents in the control condition (against the 95 in the ACt condition). After this selection the univariate analyses were run again. Now there were no significant differences left among the continuous variables, with the smallest p-value being .14, and the multivariate analysis including all continuous baseline variables together showing a F-value below 1 and a p-value of .80. Also with regard to the two categorical variables (level of education and gender), the condition did not differ significantly (p-values > .11). The conclusion is that smokers in the ACt condition and the control condition are similar now and are comparable (Table 1).

**Table 1:** **Baseline comparability of the smokers in the ACt condition and in the control condition after applying the matching procedure in respondents**

Variable	ACt condition	Control condition	p-value
Gender, female	50.9%	40%	.12
Level of education			.51
Low	25.3%	26.3%	
Medium	38.9%	31.6%	
High	35.8%	42.1%	
	M (SD)	M (SD)	
Age	44.4 (8.59)	45.4 (14.1)	.55
Number of cigarettes	16.5 (6.57)	15.9 (8.97)	.58
FTND	3.87 (2.09)	3.74 (2.37)	.66
Pros of quitting			
Long term	2.18 (0.82)	2.16 (0.84)	.86
Short term	2 (0.67)	1.99 (0.69)	.91
Social	1.79 (0.94)	1.62 (0.97)	.20
Self-evaluative	1.66 (0.67)	1.69 (0.82)	.83
Pros of smoking			
Cognitive	3.44 (0.73)	3.31 (0.75)	.22
Affective	3.87 (0.76)	3.75 (0.77)	.26
Self-efficacy situations			
Social	4.56 (1.28)	4.31 (1.23)	.14
Emotional	4.18 (1.39)	4 (1.47)	.38
Habitual	4.93 (1.3)	4.66 (1.41)	.16

Note: Due to missing values, the number of smokers in the ACt condition varied from 93 to 95 and in the control condition from 112 to 114. Univariate analysis of variance was used to test condition differences; only for gender and level of education Chi-square analyses were applied.

#### Main analyses in respondents

The smokers in the ACt condition (n = 95) and the above selected smokers in the control condition (n = 114) were included in the main analyses of testing the difference between both conditions on the self-reported continuous abstinence after 13 months. The percentages of smokers reporting continuous abstinence were 41.1% in the ACt condition and 9.6% in the control condition. To test whether this difference was significant, a logistic regression analysis with condition as the central predictor and continuous abstinence as dependent variable was conducted. This analysis showed that in the ACt condition significantly more smokers were abstinent, Exp(B) = 6.52, 95% CI 3.1-13.72; p < .001. To further ascertain that the 14 variables that were used to compare the conditions on were not responsible for the significance, the same analysis was run in the same matched selection, now controlling for these 14 variables. The effect of condition remained significant, Exp(B) = 9, 95% CI 3.67-22.07; p < .001.

### All-cases analyses

#### Comparability and matching

In the all-cases analyses, all drop outs were considered to not be abstinent at T3 [19]. This led to percentages of abstinence in the ACt condition (n = 124) of 31.5% and in the control condition (n = 161) of 7.5%. Next, the baseline comparability of the smokers in both conditions in this sample was investigated. When comparing both conditions on the 14 variables it seemed that smokers in the control condition scored significantly higher on affective pros of smoking, and significantly lower on self-efficacy in social situations, as well as on social pros of quitting. To increase the comparability of the control condition, the smokers with the lowest 10% scores on the affective pros, social self-efficacy and social pros were removed, leaving 121 T1 smokers in the control condition. The percentage of abstinence in the ACt condition remained 31.5%, against 8.3% in the control condition.

#### Main analyses all-cases

The logistic regression analysis with condition as the central predictor and continuous abstinence as dependent variable shows that in the ACt significantly more smokers were abstinent, Exp(B) = 5.09, 95% CI 2.41-10.78; p < .001. To ascertain that the above variables in the comparability analyses were not responsible for the significance, the same analysis was run in the same matched selection, now controlling for these variables. The effect of condition remained significant, Exp(B) = 5.78, 95% CI 2.5-13.38; p < .001.

### CO-validated results

#### Preparation

Of the 95 smokers in the ACt condition, 39 reported continuous abstinence at T3. The aim was to verify these self-reports with a CO-validation measure. The actual validation procedure took place between 1 and 4 weeks after the self-report of abstinence. We were able to conduct the validation procedure in 30 of the 39 self-reported abstinent respondents in the ACt. Of the nine missing, three could not be contacted because they were on holiday, three could not be scheduled at a work location that we visited (for example, because they drove a truck and had no office), two did not respond to our e-mails or telephone calls, and one reported that he started smoking again after the self-report of abstinence some weeks ago.

Of the 30 that followed the validation procedure, one person reported right beforehand to have started to smoke again after the self-report of abstinence some weeks ago. In the remaining 29 respondents, all CO-scores were 7 ppm or lower: two respondents had a CO level of 7 ppm, three had a level of 6 ppm, and all others had levels of 5 ppm and lower. Thus, in these 29 respondents who (still) claimed abstinence, abstinence was verified using the cut-off point of 10 ppm.

#### Main analyses CO validated abstinence

The above reported logistic regression analyses to test the differences in proportions of abstinent smokers were now repeated in the matched groups of respondents while treating the 9 self-reported abstinent respondents who could not be CO validated as smokers (ACt condition n = 95; control condition = 114). Thus, for this purpose their self-report of abstinence was considered to be false.

Analyses in the respondents now showed 31.6% abstainers in the ACt condition and 9.6% in the control condition, while the logistic regression analysis showed that the difference was significant, Exp(B) = 4.73, 95% CI 2–11.19; p < .001. The all-cases analysis now showed percentages abstinence in the ACt and the control condition (ACt condition n = 124; control condition = 161) of 24.2% and 7.5%, respectively. The logistic regression analysis still showed a significant difference, Exp(B) = 3.81, 95% CI 1.72-8.47; p = .001. Including the covariates in these logistic regression analyses did not change the levels of significances.

## Discussion

The present quasi-experimental design showed whether it is fruitful to expose smokers “in companies to the ACt”. Thus, the treatment package of “ACt in companies” was tested. All statistical analyses on the differences between both conditions on the percentages of self-reported continuous abstinence were significant, even the most conservative. The percentage continuous abstinence in the ACt condition ranged from 24.2% to 41.1% depending on the assumptions and on the selections of participants, and ranged from 7.5% to 9.6% in the control condition. When the Exp(B) is interpreted as an Odds Ratio, smokers following the ACt in their company were about 6 times more likely to have quit smoking after 13 months compared to similar smokers in the general population.

The value of the results crucially depends on the comparability of the smokers in both conditions. When the smokers were not similar, differences in smoking cessation might be caused by these existing differences and not by the ACt. To study comparability, we included three types of variables that might be relevant for smoking cessation: Demographic variables, smoking behavior, and psychological determinants of smoking cessation. The main analyses were significant when these variables were taken into account. Therefore, the next question is: “Did we miss any differences between the smokers in both conditions that might be responsible for the effect?”. In other words, is it plausible that one or more not detected differences between the smokers in the conditions can explain the differences in percentages abstainers?

Not all possible smoking and quitting history variables were assessed. The number of past quit attempts and the duration of the longest quit attempt were not assessed although they are related to smoking cessation success [20]. However, one pathway through which past behavior influences future behavior is through shaping people’s perception regarding the behavior [21]. The most important perceptions regarding smoking cessation were taken into account in our study in the pros of smoking, the pros of quitting and self-efficacy. A measure of smoking history that was not assessed was the number of years smoked, as a proxy of the duration of the experience with smoking. However, because most smokers start to smoke regularly between the ages of 14 and 18 [22–24], age and number of years smoked are strongly associated. Therefore, statistically controlling for age should have largely controlled for the number of years smoked. Also, the age of starting to smoke was not assessed. A large survey among youngsters [25] suggests that those who start at younger age are more likely to become regular and heavy smokers. However, we argue that in our analyses regularity and heaviness of smoking was taken into account by the measures of number of cigarettes smoked a day and the FTND scores. All in all, it cannot be completely ruled out here that differences between the conditions in smoking history and quitting history influenced the results.

A psychological variable that was not included was the intention to quit: It may have been that smokers in the ACt and the control group differed in intention to quit. We omitted intention for the following reason: Within the context of the ACt being offered to smokers in companies, with its resulting constraints, the pretest measurement could only be conducted in the ACt participants after they were informed that they would actually join the ACt. This anticipation especially would have biased assessments of the readiness or intention to quit, disturbing the actual comparability with the control group. However, we did assess the motivation to quit with a well validated measure (with the pros of quitting scales) that includes different aspects of the motivation and that is probably not susceptible to such a bias (i.e., smokers are not expected to see more positive effects of quitting when they know they would join the ACt). The conditions were similar on this baseline measure.

In conclusion, the most relevant variables were taken into account in comparing the similarity of the ACt smokers and the smokers in the control condition, and we think that it is plausible that the most obvious difference between the conditions that is designed and expected to lead to abstinence - the ACt - is responsible for the large or robust differences in percentage of abstinence. Moreover, almost 30% of the respondents in the control condition indicated to have received smoking cessation support, including nicotine replacement, coaching or self-help materials. This suggests that smokers in the control condition were also engaged in quitting, and were not only interested in joining research or receiving the financial rewards. However, we cannot rule out that having agreed to attend a specific smoking cessation intervention (i.e., the ACt) has contributed to the outcomes in the ACt condition. Thus, some uncertainty remains about the similarity and, therefore, about the effectiveness of the ACt.

It is unclear what exactly may have caused the smokers to quit in the ACt. It is plausible that the one-session expectancy challenge, which comprises the core of the ACt, caused smokers to quit: The expectancy challenge has been shown to have change potential in lowering alcohol use [6–8]. However, other aspects of the package of “ACt in companies” that might be responsible for the effects are the training taking place: 1) in a company context and; 2) in groups. With regard to the latter, a recent (uncontrolled) study on the effects of a one-session large group smoking cessation intervention of different content - cognitive behavior therapy plus pharmacotherapy advice - showed promising results [26]. This suggests that the one-session format can be sufficient to induce change. Future studies might dismantle the treatment package that was tested in the present study.

One relevant shortcoming of the CO-validation is that it only can reliably assess smoking during the past 24 hours, while the self-reported continuous abstinence that it was supposed to validate referred to having refrained from smoking for over a year. Future studies might want to further validate such long term continuous abstinence self-reports, although at present there is no ideal way to do so.

## Conclusions

It is plausible that the differences in percentages abstainers in the conditions can be attributed to the ACt provided in companies. Moreover, in our view it is less plausible that the large or robust differences can be explained by factors we did not assess. In addition, the expectancy challenge strategy that comprises the main ingredient of the ACt has been shown to be effective in reducing alcohol consumption, strongly suggesting that it has potential to induce behavior change. In conclusion, the present study reveals relevant clues that providing smokers with the package of ACt in companies can be an effective smoking cessation strategy, although uncertainties inherent to the quasi-experimental design remain.

## Abbreviations

ACt: Allen Carr training
CO: Carbon Monoxide
RCT: Randomized Controlled Trial.
